# The impact of depression on the quality of life of lung cancer patients undergoing chemotherapy: mediating effects of perceived social support

**DOI:** 10.3389/fpsyt.2025.1526217

**Published:** 2025-03-21

**Authors:** Fan Xu, Shaoju Xie, Qiao Li, Xiaoli Zhong, Jiquan Zhang

**Affiliations:** ^1^ Oncology Department, Deyang People’s Hospital, Deyang, Sichuan, China; ^2^ Nursing Department, Deyang People’s Hospital, Deyang, Sichuan, China; ^3^ Nephrology Department, Deyang People’s Hospital, Deyang, Sichuan, China

**Keywords:** lung cancer, depression, perceived social support, quality of life, mediation effect

## Abstract

**Background:**

Quality of life (QOL) in patients undergoing chemotherapy for lung cancer has been a key research area. Numerous studies have examined the relationships among depression, perceived social support (PSS), and QOL. However, the mechanisms underlying PSS in lung cancer patients receiving chemotherapy remain underexplored.

**Objective:**

To investigate the mediating role of PSS in the relationship between depression and QOL in lung cancer patients undergoing chemotherapy.

**Methods:**

A convenience sample of 390 lung cancer patients undergoing chemotherapy was selected from the outpatient clinics and wards of the Department of Oncology at a tertiary hospital in Deyang City between January 2024 and June 2024. Participants completed a general information questionnaire, Self-Rating Depression Scale (SDS), Multidimensional Scale of Perceived Social Support (MPSSS), and Functional Assessment of Cancer Therapy-Lung Cancer (FACT-L) scale. SPSS 26.0 was used to analyze correlations between depression, PSS, and QOL, while AMOS 26.0 assessed the mediating effect of PSS on the relationship between depression and QOL.

**Results:**

The mean scores for depression, PSS, and QOL were 39.79 ± 11.63, 68.96 ± 13.09, and 66.43 ± 23.67, respectively. Pearson’s correlation analysis showed that depression was negatively correlated with QOL (r = -0.319, *P* < 0.001) and positively correlated with PSS (r = -0.484, *P* < 0.001). Additionally, PSS was positively correlated with QOL (r = 0.349, *P* < 0.001). PSS partially mediated the relationship between depression and QOL, with a mediating effect value of -0.165, accounting for 50.3% of the total effect.

**Conclusion:**

Depression in lung cancer patients undergoing chemotherapy directly impacts QOL and indirectly affects it through PSS. Clinically, healthcare providers should address depression in these patients and offer psychological support and interventions as needed. Additionally, medical institutions can implement targeted strategies to help patients build a strong social support system, reduce negative emotions, enhance psychological well-being, and improve overall QOL.

## 1 Introduction

Global cancer statistics for 2022 indicate that the number of new lung cancer cases worldwide reached 2.48 million, accounting for 12.4% of all new cancer cases, making it the most common cancer globally (1). It is noteworthy that this large group of patients generally faces a serious psychosocial burden. Due to the insidious onset of lung cancer, the lack of effective screening methods, and nonspecific early-stage symptoms, many patients are diagnosed at advanced stages. The age-standardized 5-year relative survival rate for lung cancer remains low, ranging from 10% to 20% in most regions (2). Chemotherapy is widely used in lung cancer treatment, offering potential benefits in prolonging survival and controlling recurrence and metastasis. However, long-term chemotherapy can lead to various adverse reactions, with prolonged physical and emotional suffering, often resulting in negative emotions that significantly impact recovery, prognosis, and quality of life (QOL) in later stages (3, 4). Thus, it is essential to focus on the psychological state and QOL of patients undergoing chemotherapy for lung cancer.

### 1.1 Depression and QOL

The World Health Organization (WHO) defines QOL as an individual’s perception of their position in life within the context of the culture and value systems in which they live, in relation to their goals, expectations, standards, and concerns. This definition encompasses an individual’s experience of their physical condition, mental health, social relationships, and overall personal circumstances (5, 6). Depression is commonly characterized by persistent low mood, loss of interest in daily activities, and reduced energy and concentration (7). Severe depressive symptoms are frequently observed in lung cancer patients. Survey data reveal that the incidence of depression among lung cancer patients is approximately 31.0%–58.1% (8–11). Without timely intervention, depression can adversely affect immune, endocrine, and neurological functions, leading to declines in physical, emotional, and social functioning, worsening symptoms, poor clinical outcomes, and diminished QOL across multiple dimensions (12–14). Numerous studies have shown a strong relationship between depression and QOL. For instance, research on medical students found that depression was negatively correlated with QOL scores, especially among those with depressive symptoms, where lower QOL scores were strongly associated with greater severity of depression (15). Another study identified depression as a significant factor reducing QOL among hospitalized older adults with pneumoconiosis (16). A recent study of older Chinese adults also reported a substantial negative impact of depression on QOL (17).

### 1.2 Depression and PSS

Social support encompasses various forms of assistance, including material, emotional, respect, informational, and peer support. This support can be classified as subjective or objective, with perceived social support (PSS) falling under subjective support. PSS refers to an individual’s emotional experience and satisfaction in feeling supported, respected, and understood by society, particularly perceived support from family, friends, and others, emphasizing self-perception and understanding of social support (18, 19). Coyne’s interpersonal theory of depression suggests that depressed individuals engage in interactions that are aversive and lack social skills (20). Certain interpersonal traits in depressed individuals—such as reduced social motivation, impaired social skills, and limited emotional expression—have been identified as factors contributing to poor social functioning (21–23). Research has shown a negative association between depression and PSS. A study of 910 community-dwelling older adults found that depressive symptoms negatively impacted PSS (24). A longitudinal study using structural equation modeling revealed that depression contributes to social support erosion (25). Additionally, research on patients with systemic lupus erythematosus (SLE) demonstrated that negative emotions, including depression and anxiety, were significantly negatively correlated with PSS, and that higher levels of negative emotions are associated with lower levels of PSS (26).

### 1.3 PSS and QOL

The main effects model of social support suggests that social support provides a general benefit, enhancing individuals’ physical and mental health regardless of their current support levels (27). Meanwhile, Social Cognitive Theory (SCT) posits that an individual’s behavior is shaped by observing others, interacting with the environment, and reflecting on these interactions (28). Within this framework, social support positively impacts QOL by enhancing self-efficacy, emotional regulation, and social networks, thus promoting positive behavior. Related studies have also shown a positive association between PSS and QOL. For instance, a study of 150 patients with cardiovascular disease found that PSS was a key strategy for improving QOL and coping with illness (29). Another study reported a positive association between PSS and QOL among migraine patients (30).

### 1.4 The mediating role of PSS

Lung cancer chemotherapy patients not only have to cope with the psychological impact of a malignant tumor diagnosis, but also have to endure multidimensional pressures such as physiological discomforts (e.g., fatigue, nausea, etc.), social role changes (e.g., decreased work ability), and increased financial burden caused by chemotherapy. The social support buffer effect model proposes that social support mitigates the harmful effects of stress when individuals encounter challenging situations (31, 32). In addition, the effects of PSS on mental health are not limited to the individual level, but may also be mediated through group-level psychological mechanisms: Pagliaro et al. (33)demonstrated that Group-Based Resiliency (GBR) indirectly enhances individual well-being by strengthening group identity and collective self-esteem when the group is facing an external threat, which suggests that in a group of cancer patients, PSS may improve their depression through similar pathways (e.g., strengthening patients’ identity with a supportive group), thereby improving their QOL. Research has shown that social support can buffer the relationship between stress and QOL (34). A study on community-dwelling older adults found that social support mediated the relationship between anxiety, depressive symptoms, and QOL, reducing the negative effects of anxiety and depression on QOL (35). Additionally, research on older patients with chronic illnesses indicated that social support mediates the relationship between depression and QOL, serving a protective role in mental health by reducing the adverse psychological impact of stressors, such as depression, and consequently improving physical and mental health (36).

Based on these findings, we proposed the following hypotheses:

Hypothesis 1: Depression has a direct effect on QOL.
Hypothesis 2: PSS mediates the relationship between depression and QOL.

### 1.5 The current study

We conducted this study to address certain limitations in prior research on QOL in lung cancer patients undergoing chemotherapy. First, previous studies primarily focused on health-related QOL (37–40), there remains a notable paucity of research employing comprehensive assessment frameworks to evaluate overall QOL as a multidimensional construct encompassing physical, psychological, and social domains. Second, while the relationships between depression, PSS, and QOL have been explored in various specific groups—such as myocardial infarction patients (41), HIV-positive individuals (42), and adolescents (43), but the social support system of patients undergoing chemotherapy for lung cancer is special. Chemotherapy - induced alopecia, altered body image, and other side effects may reduce patients’ willingness to engage in social activities. Moreover, social isolation due to frequent hospitalization may further impair PSS. To date, no studies have specifically examined these relationships in lung cancer patients undergoing chemotherapy. Third, the mechanisms underlying the association between depression and QOL remain insufficiently understood. The role of PSS as a stress - coping resource, which may function through a dual pathway - both buffering the direct negative impact of depression on QOL(stress - buffering effect) and promoting adaptive behaviors (e.g., increased treatment adherence) through enhanced self - efficacy - remains an under - evaluated and under - researched topic.

**Figure 1:**
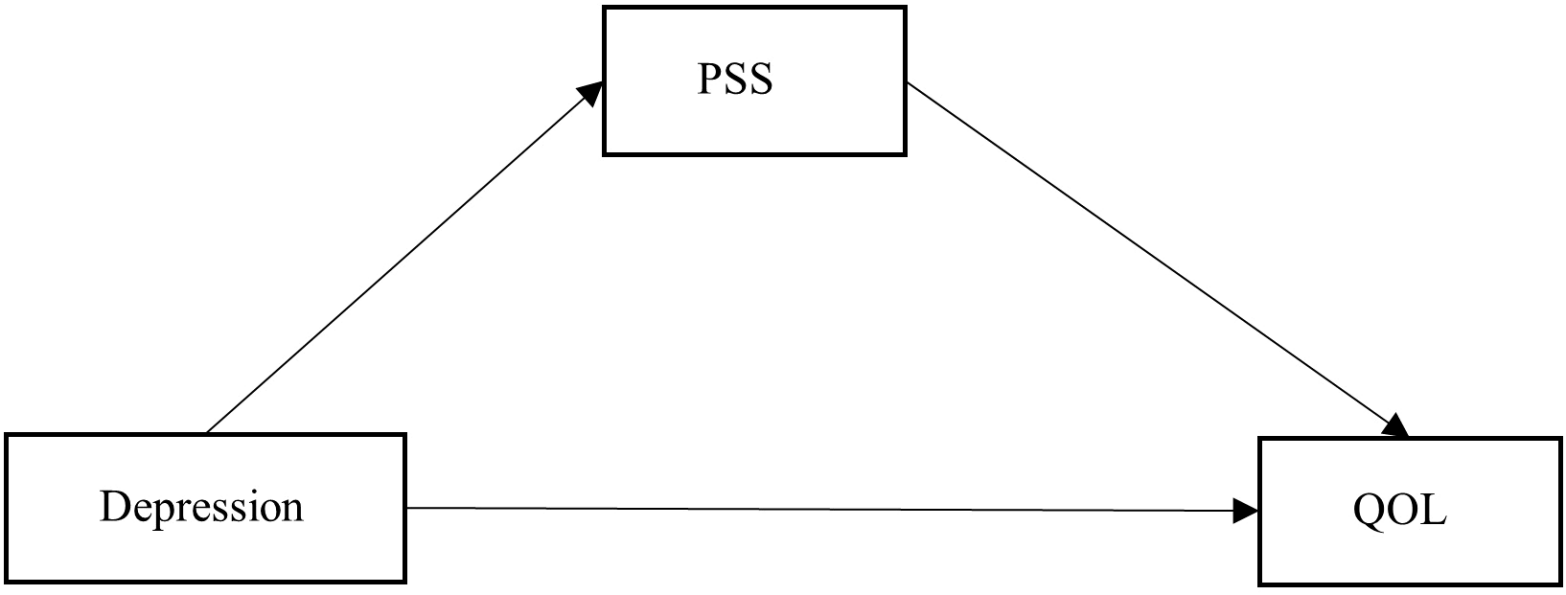
Hypothesized theoretical model.

In summary, guided by the buffer effect model and main effect model of social support, this study formulates specific research hypotheses. The hypothesized model developed for this study is presented in **Figure 1**. This study aimed to: (1) investigate the relationship between depression and QOL in lung cancer patients undergoing chemotherapy; and (2) confirm the mediating role of PSS in the relationship between depression and QOL.

## 2 Materials and methods

### 2.1 Study design and participants

A cross-sectional design was employed. Subjects were recruited between January and June 2024 at a tertiary hospital in Deyang City. The researcher distributed the general information questionnaire, SDS, MPSSS, and FACT-L scale to lung cancer patients undergoing chemotherapy, with completion taking 20-30 minutes.

### 2.2 Participants

Inclusion criteria were: (1) patients diagnosed with primary bronchopulmonary cancer and admitted for chemotherapy after histopathologic confirmation; (2) consciousness and no communication barriers; (3) age ≥18 years; (4) expected survival time ≥6 months; and (5) voluntary participation. Exclusion criteria included: (1) scheduled surgery post-diagnosis; (2) mental or cognitive disorders (e.g. schizophrenia, bipolar disorder, Alzheimer’s disease, etc.); (3) lack of awareness of their cancer diagnosis; and (4) comorbidities involving serious heart, liver, kidney, or other organ diseases.

The sample size was calculated using the formula n=[u_α_*σ/δ]² for cross-sectional surveys, with α=0.05 on both sides, corresponding to u_α_ =1.96, tolerance error δ = 2.0, Standard deviation σ= 15.554 was set based on a study of 625 lung cancer patients in mainland China investigating QOL using the same scale (FACT-L) (44). This yielded a minimum of 232 participants; accounting for a 20% attrition rate, the target sample was 255. The study included 390 participants.

### 2.3 Ethics statement

This study was approved by the Ethics Committee of Deyang People’s Hospital (202204058K01). Prior to participation, a trained investigator explained the study’s purpose, significance, and survey instructions to patients. Informed consent was obtained, and surveys were completed anonymously to ensure confidentiality.

### 2.4 Measures

#### 2.4.1 Demographic characteristics and disease-related information

A general information questionnaire collected demographic and disease-related data, including age, gender, number of children, marital status, education, religion, occupation, monthly household income, residence, cancer stage, and comorbidities. Indicator categorization is shown in **Table 1**.

**Table 1:** Demographic characteristics and disease-related information of patients undergoing chemotherapy for lung cancer.

Variable		N (%)	QOL (Mean ± SD)
Gender	Male	282(72.31)	65.72 ± 24.16
	Female	108(27.69)	68.31 ± 22.34
	*t*		-0.966
	*P*		0.334
Age(Year)	18~44	23(5.90)	77.13 ± 23.34
	45~59	183(46.92)	67.86 ± 23.15
	≥60	184(47.18)	63.67 ± 23.85
	*t*		3.992
	*P*		**0.019**
Number of children	0	4(1.03)	68.50 ± 25.44
	1	255(65.38)	66.98 ± 23.02
	2	95(24.36)	64.18 ± 24.41
	≥3	36(9.23)	68.25 ± 26.61
	*F*		0.412
	*P*		0.744
Marital status	Married	326(83.59)	66.44 ± 23.22
	Single	28(7.18)	66.32 ± 29.40
	*t*		0.026
	*P*		0.989
Education level	Primary and below	152(38.97)	65.26 ± 26.33
	Middle school	199(51.03)	66.12 ± 22.06
	College and above	39(10)	72.62 ± 20.05
	*F*		1.539
	*P*		0.216
Religion	Yes	23(5.9)	66.00 ± 19.95
	No	367(94.1)	66.46 ± 23.91
	*t*		-0.090
	*P*		0.928
Occupational status	Employed	57(14.62)	63.42 ± 24.80
	Retired	169(43.33)	68.94 ± 22.55
	Unemployed	164(42.05)	64.90 ± 24.29
	*F*		1.762
	*P*		0.173
Per capita monthly household income(Yuan)	<3000	232(59.49)	64.38 ± 23.23
	3000-4999	85(21.79)	65.02 ± 24.06
	5000-9999	54(13.85)	71.02 ± 20.21
	≥10000	19(4.87)	84.79 ± 28.33
	*F*		5.338
	*P*		**0.001**
Place of residence	City	221(56.67)	67.78 ± 23.62
	Townships	48(12.31)	66.65 ± 24.85
	Villages	121(31.03)	63.88 ± 23.29
	*F*		1.063
	*P*		0.347
Clinical stage of cancer	I	25(6.41)	76.24 ± 29.32
	II	85(21.79)	71.20 ± 26.40
	III	177(45.38)	66.28 ± 21.03
	IV	103(26.41)	60.38 ± 22.77
	*F*		4.975
	*P*		**0.002**
Comorbidity with other diseases	Yes	218(55.90)	64.85 ± 21.64
	No	172(44.10)	68.44 ± 25.95
	*t*		1.459
	*P*		0.145

Boldface indicates statistically significant differences (p < 0.05).

#### 2.4.2 Measurement of QOL

The Functional Assessment of Cancer Therapy-Lung Cancer (FACT-L) scale, developed by Cella (45) and adapted to Chinese by Wan (46), assesses QOL in Chinese lung cancer patients (47). The scale includes 36 items across five dimensions: Physical Well-Being (PWB), Social/Family Well-Being (SWB), Emotional Well-Being (EWB), Functional Well-Being (FWB), and the Lung Cancer Subscale (LCS), Such as "I have pain","I’m afraid of death","I have a cough". Each item is scored on a 5-point Likert scale (0-4), for a total score of 0-144, with higher scores indicating better QOL. The scale’s Cronbach’s alpha was 0.758, and in this study, it was 0.926.

#### 2.4.3 Measurement of depression

The Self-Rating Depression Scale (SDS), developed by Zung (48) in 1965, measures depression severity. and the Cronbach’s alpha coefficient was 0.900 when applied to lung cancer patients in China, which is suitable for assessing the depression status of lung cancer patients in China (49).The 20-item scale covers four dimensions: psychotic affective symptoms (PAS), somatic disorders (SD), psychomotor disorders (PD), and depressive mental disorders (DMD), rated on a 4-point Likert scale (1-4), Such as"I feel emotionally frustrated and depressed","I don’t sleep well at night","I feel weight loss". Items are scored such that higher total scores (20-80) indicate more severe depression. The scale’s Cronbach’s alpha in this study was 0.867.

#### 2.4.4 Measurement of PSS

The Multidimensional Scale of Perceived Social Support (MPSSS), developed by Zimet (18) and translated by Jiang (50), measures PSS from family and significant others, and emphasizes an individual’s subjective feelings and experiences of social support rather than relying solely on objective social support resources. objective social support resources. The Cronbach’s alpha coefficient of this scale when applied to lung cancer patients in China was 0.949, and the Cronbach’s alpha coefficients of each dimension were 0.930, 0.932, and 0.891, respectively (51).It comprises three dimensions: family support (FAS), friend support (FRS), and significant other support (SOS), across 12 items, Such as"My family has been able to help me in concrete ways","I My friends can really help me.","My friends can share my joys and sorrows". Responses are rated on a 7-point Likert scale (1-7), with total scores ranging from 12 to 84; higher scores reflect greater PSS. The Cronbach’s alpha for this study was 0.924.

### 2.5 Data analysis

SPSS 26.0 was used for data analysis. PP plots and histograms tested the approximate normality of the data distribution. Measurements are described as mean ± standard deviation, and counts are presented as the number of cases and constitutive ratios. Differences in QOL between categorical groups were tested using t-tests and one-way analysis of variance (ANOVA), with *post-hoc* analyses performed by the Least Significant Difference (LSD) method. Pearson correlation analysis tested the relationships between depression, PSS, and QOL, and Fisher transformations were used to calculate their confidence intervals. Common method bias was assessed using exploratory factor analysis in Harman’s one-way test, and structural equation modeling of influence pathways was conducted with AMOS 26.0. The significance of mediating effects was tested using the bias-corrected percentile Bootstrap method, with α = 0.05.

## 3 Results

### 3.1 Demographic characteristics and disease-related information

**Table 1** presents the demographic characteristics and disease-related information of lung cancer patients undergoing chemotherapy. The sample included 390 patients, with a mean age of 59.11 years (SD = 11.37; range 28–83); 72.31% were male, 47.18% were aged ≥60 years, 65.38% had one child, 83.59% were married, and 38.72% had an education level of junior high or secondary school. Additionally, 94.1% of patients had no religious beliefs, 43.33% were retired, 59.49% had a per capita monthly family income below 3,000 yuan, 56.67% resided in urban areas, 46.92% had stage III cancer, and 55.9% had comorbid diseases.

Significant differences in QOL were observed across age[F(2, 387) = 3.992, *P* = 0.019, η²= 0.020], per capita monthly household income[F(3, 386) = 5.338, *P* = 0.001, η²= 0.040], and cancer clinical stage[F(3, 386) = 4.975, *P* = 0.002, η²= 0.037]. *Post hoc* tests revealed that patients aged 18–44 had higher QOL scores than those aged ≥60 [MD = 13.457, 95% CI (3.24, 23.67), *P* < 0.05]. Patients with a per capita monthly income above 10,000 yuan had higher QOL scores than those in other income groups: compared with less than 3000 [MD = 20.410, 95% CI (9.48, 31.34), *P* < 0.05], with 3000-4999 [MD = 19.766, 95% CI (8.15, 31.38), *P*< 0.05], and with 5000-9999 [MD = 13.771, 95% CI (1.56, 25.98), *P*< 0.05];Patients with stage IV cancer had lower QOL scores than those at other stages: Compared with phase I [MD = -15.861, 95% CI (-26.08, -5.64), *P*< 0.05], with phase II [MD = -10.821, 95% CI (-17.54, -4.10), *P*< 0.05], and with phase III [MD = -5.904, 95% CI (-11.59, -0.22), *P*< 0.05], and stage III patients had lower QOL scores than stage I patients [MD = -9.958, 95% CI (-19.75, -0.16), *P*< 0.05].

### 3.2 Correlation analysis of depression, PSS and QOL in lung cancer chemotherapy patients

Correlations, means, and standard deviations of the related variables are presented in **Table 2**. Correlation analysis revealed that the total depression score in lung cancer chemotherapy patients was negatively correlated with the total PSS score (r = -0.484, *P* < 0.01) and negatively correlated with the total QOL score (r = -0.319, *P* < 0.01). Additionally, the PSS score was positively correlated with the total QOL score (r = 0.349, *P* < 0.01).According to Cohen’s criterion, the r-values all fall within the range of | 0.3 | to | 0.5 |, all of which are moderately correlated (52).as shown in **Table 2**.

**Table 2:** Correlation analysis of depression, PSS and QOL in lung cancer chemotherapy patients.

Variable	Mean	SD	Depression	PSS	QOL
Depression	39.79	11.63	1		
PSS	68.96	13.09	-0.484^**^	1	
QOL	66.43	23.67	-0.319^**^	0.349^**^	1

^**^ *P* < 0.01.

### 3.3 Analysis of the mediating effect of PSS between depression and QOL in lung cancer chemotherapy patients

An exploratory factor analysis using the Harman’s one-way test was conducted by including all entries of depression, PSS, and QOL in this study, revealing a total of 14 common factors with eigenvalues >1. The variance explained by the first common factor was 19.95%, below the critical threshold of 40% (53), indicating no significant common methodological biases in the data.

A structural equation model was constructed in AMOS 26.0, with depression as the independent variable, PSS as the mediator, and QOL as the dependent variable. The maximum likelihood method was applied to estimate the parameters. The initial model showed poor fit indices(χ^2^/df=3.363, RMSEA=0.078, IFI=0.946, GFI=0.938, NNFI=0.946, CFI=0.946, NFI=0.925). As the chi-square degrees of freedom ratio exceeded 3, following the principle of Modification Index (MI) from the largest to the smallest, the double-arrow correlation paths of e11 and e14 were added to the model to correct the model, and the corrected model fits better (χ^2^/df=1.822, RMSEA=0.046, IFI=0.982, GFI=0.963, NNFI=0.976, CFI=0.982, NFI=0.960), all within acceptable limits (54),the fit indices and reference standards of the model before and after correction were shown in **Table 3**.

**Table 3:** Fit indices and reference standards of the model before and after correction.

Fit indices	*x* ^2^ */df*	*RMSEA*	*IFI*	*GFI*	TLI	*CFI*	*NFI*
Reference standards	<3.0	<0.08	>0.90	>0.90	>0.90	>0.90	>0.90
Pre-correction	3.363	0.078	0.946	0.938	0.946	0.946	0.925
Post-correction	1.822	0.046	0.982	0.963	0.976	0.982	0.960

*x* ^2^ */df*, chi-square (math.) degree of freedom ratio; RMSEA, Root Mean Square Error of Approximation; IFI, Incremental Fit Index; GFI, Goodness of Fit Index; TLI, Tucker Lewis Index; CFI, Comparative Fit Index; NFI, Normed Fit Index.

**Figure 2:**
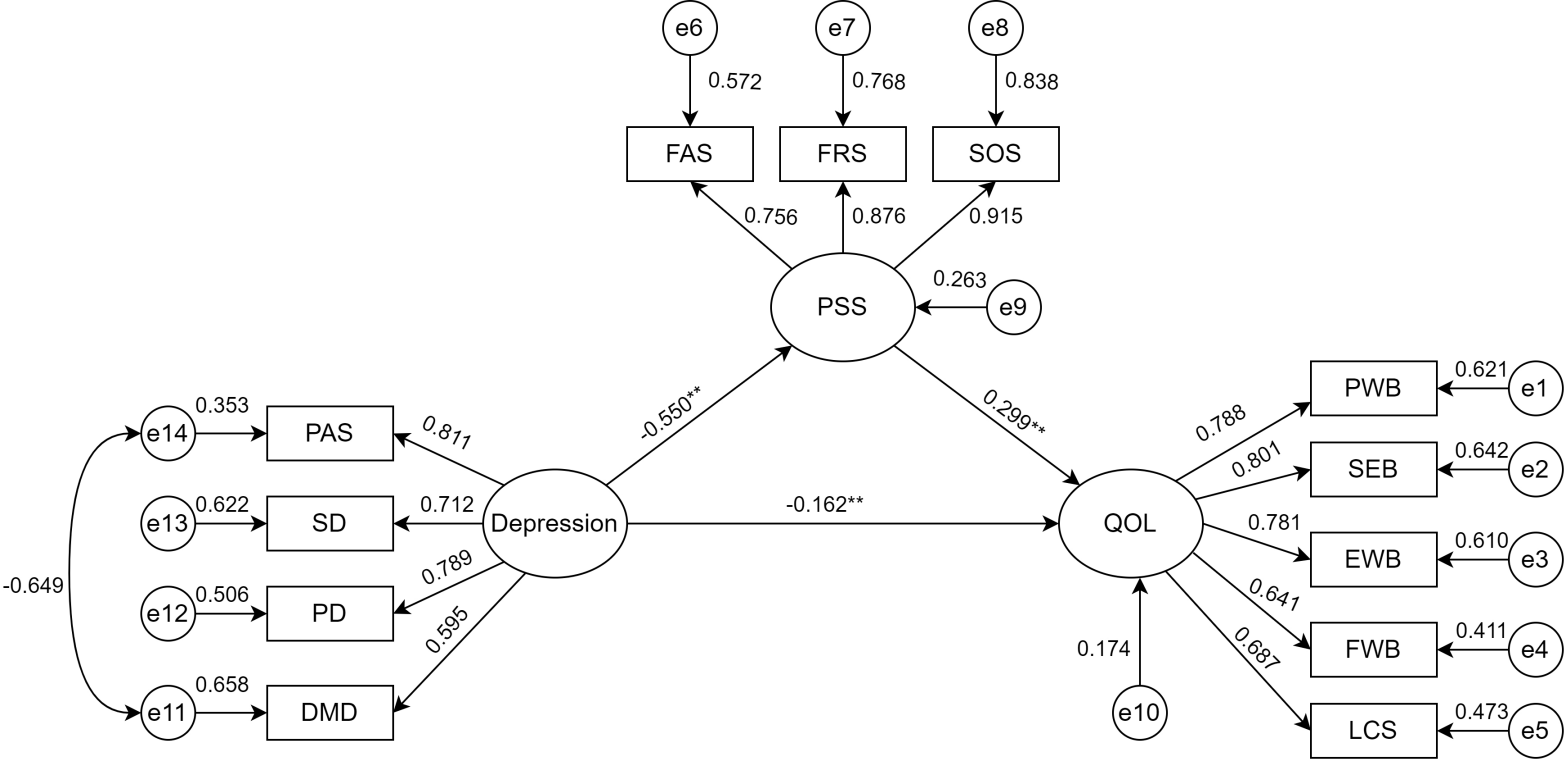
Modeling the mediating effect of depression on QOL through PSS. ^∗∗^Indicating the coefficient of the path is significant(*P*<0.01). PAS, psychotic affective symptoms; SD, somatic disorders; PD, psychomotor disorders; DMD,depressive mental disorders; PSS, perceived social support; FAS, family support; FRS, friend support; SOS, significant other support; QOL, quality of life; PWB, physical well-being; SWB, social/family well-being; EWB, emotional well-being; FWB, functional well-being; LCS, lung cancer subscale.

A bootstrap test of the model’s mediating effect was performed with 5000 samples and a 95% confidence interval (55). The standardized total effect of depression on QOL was -0.326 (95% CI: -0.432 to -0.206). The standardized direct effect of depression on QOL was -0.162 (95% CI: -0.303 to -0.010), accounting for 49.7% of the total effect. The standardized indirect effect of PSS between depression and QOL was -0.165 (95% CI: -0.247 to -0.094), representing 50.3% of the total effect. These results indicate that PSS partially mediates the relationship between depression and QOL (**Figure 2**, **Table 4**).

**Table 4:** Mediating effects of PSS between depression and QOL in lung cancer chemotherapy patients.

	Effect	BootSE	*95%CI*	Ratio of effect
Total path of X on Y	-0.326	0.059	-0.432 ~ -0.206	–
Direct path of X on Y	-0.162	0.074	-0.303 ~ -0.010	49.7%
Indirect path of X on Y	-0.165	0.039	-0.247 ~ -0.094	50.3%

X, depression; Y, quality of life.

## 4 Discussion

This study was conducted based on Hypothesis 1 and 2, which confirmed the direct effect of depression on QOL in lung cancer chemotherapy patients and provides preliminary validation that PSS mediates the relationship between depression and QOL. These findings suggest new approaches to improve QOL in lung cancer chemotherapy patients by addressing both depression and PSS, which provide new perspectives for improving patients’ QOL, suggesting that we should pay attention to patients’ depression while should also emphasize the construction and strengthening of their social support system.

Among demographic and disease-related factors, age, monthly household income per capita, and cancer clinical stage were significant predictors of QOL. Age negatively impacted QOL, consistent with previous studies (56). This may be because, with age, individuals experience declines in health status and material resources, affecting their QOL. Monthly household income had a positive effect on QOL, as studies on cancer patients indicate that economic stability is a protective factor for QOL (57, 58). Higher income allows patients to afford better medical care, reducing financial stress and supporting recovery. Conversely, patients with lower incomes face greater financial pressure, which can increase physical and mental burdens and diminish QOL. The clinical stage of cancer also negatively affected QOL, aligning with prior research that correlates advanced cancer stages with greater symptom burden and lower QOL (59). Patients with earlier-stage disease tend to have milder symptoms, minimal impact on self-care abilities, and fewer psychological and social disruptions. In contrast, advanced-stage disease brings more severe symptoms that significantly impair physical, psychological, and social functions, thus reducing QOL (60).

Depression was significantly and negatively associated with QOL, supporting prior findings (61, 62). Chemotherapy, as a primary treatment for lung cancer, can damage normal cells, including bone marrow and gastrointestinal mucosal cells, due to its cytotoxicity. This results in decreased immune function, appetite loss, gastrointestinal symptoms, and other side effects that compromise both QOL and psychological well-being (63, 64). Research suggests that psychological and physiological states interact; a positive psychological outlook can enhance immune response, improve body coordination and recovery potential, while a negative mindset may impede recovery (65–67). Furthermore, Studies have shown that depression lowers adherence and compliance behaviors, leading to resistance to treatment and non-cooperation, negatively affecting clinical outcomes, prognosis, and ultimately QOL (68, 69).

Depression was also significantly negatively correlated with PSS, consistent with previous findings (70). PSS reflects an individual’s sense of help and support from social interactions and is closely linked to physical and mental health (62). Cognitively, depressed individuals often focus inwardly (71), showing little interest in others (72), and this cognitive bias leads to a reduced ability to perceive the supportive intentions of others, e.g., misinterpreting other people’s concern for sympathy or charity, and consequently refusing to accept support (73). Emotionally, depression also brings low mood and negative thinking patterns (74, 75), causing individuals to feel undeserving of help during challenging times. This mindset hinders them from actively seeking or accepting support from family, friends, and healthcare providers, exacerbating feelings of loneliness and helplessness, thus creating a cycle that reduces their receptivity to social support (76). Behaviorally, symptoms associated with depression, such as diminished interest and decreased energy, directly result in reduced social activities. A study published in Nature uncovered the neural circuit mechanisms linking depressive symptoms to social avoidance behaviors using an animal model. Specifically, depressive symptoms, such as anhedonia, in depression model mice were found to lead to the development of social avoidance behaviors and a decline in social activities (77). Additionally, the relationship between depression and PSS is significantly bidirectional. Relevant studies have indicated that low levels of PSS can increase the risk of developing depression by up to 4.99 times (OR = 4.993, 95% CI: 2.823–8.830) (78). Conversely, depression can reduce an individual’s perception of social support, thereby creating a vicious cycle.

PSS showed a significant positive association with QOL, aligning with previous findings; for instance, Vivek observed that higher PSS correlated with improved QOL (41). From an emotion regulation perspective, social support activates the individual’s emotion regulation center and promotes positive cognitive restructuring, making patients more inclined to adopt problem-oriented coping strategies (79); Studies indicate that PSS and a supportive social climate help patients effectively manage disease challenges and cognitive adaptation (80), thus enhancing QOL. In term of behavior change, given humans’ inherent social nature, a supportive social environment is crucial for survival, and illness can severely disrupt positive social interactions and self-confidence (81, 82). PSS promotes health and QOL by improving self-care, medical compliance, treatment adherence, lifestyle adjustments, and increasing access to disease-related information (83, 84). In terms of increased coping resources, according to Social Cognitive Theory (SCT), PSS enhances patients’ QOL by bolstering their competence and self-efficacy, enhancing the availability of coping resources, reducing illness avoidance, aiding coping strategies, and minimizing adverse physiological responses (85, 86).A study examining PSS, coping strategies, and QOL in lung cancer patients revealed that increased PSS significantly enhances positive coping strategies, thereby improving QOL. Conversely, decreased PSS is associated with increased negative coping strategies, resulting in a decline in QOL (87).

This study not only verified the direct impact of depression on QOL but also confirmed PSS as a mediator between depression and QOL. Specifically, PSS mitigated depression’s negative impact on QOL. Facing stressful events like illness and chemotherapy, lung cancer patients often experience negative emotions, and PSS provides them with emotional and practical support, helping them manage illness-related challenges and improving QOL (88). According to the social support buffer theory, PSS acts as a protective factor, effectively easing depression’s impact on mental health. Social support, including emotional, informational, and material support, can shape an individual’s psychological perception and emotional response, promoting a positive self-view and reducing psychological burdens, which lessens depression’s impact on QOL (89).

## 5 Conclusion

Our findings affirm depression’s direct impact on QOL and underscore PSS’s mediating role between depression and QOL, a relationship not previously fully explored in lung cancer chemotherapy patients. The study’s practical implications are especially relevant for these patients. We offer the following recommendations:

1. Clinical attention to psychological status: Clinicians should focus on the psychological health of lung cancer chemotherapy patients, particularly their depression levels, providing psychological guidance and intervention to help patients manage depression and mitigate its adverse effects.
2. Educational and support initiatives: Medical institutions should organize targeted public lectures and patient support groups, encourage patients to actively participate in learning about their disease, and after the lectures, invite patients to share their experiences with those who are experiencing similar difficulties and support each other, so as to enable the patients to establish a correct perception of the disease, thus improving the patients’ confidence in overcoming the disease.
3. Emotional and social support: Family members and medical staff should provide essential emotional support to meet patients’ psychological needs. Patients should be encouraged to participate in social activities, which can expand their support network, alleviate disease-related stress and treatment side effects, and improve QOL by enhancing positive emotional and psychological outlooks.

## 6 Limitations

This study has several limitations. First, we used a cross-sectional research design that allowed us to collect data only at a specific point in time, making it impossible to determine the causal and temporal sequence between variables, and the questionnaire was self-reported for patients to complete, which prevented us from drawing directional conclusions about the relationships between depression, PSS, QOL, demographic characteristics, and disease-related variables.

Second, the study population consisted of lung cancer chemotherapy patients from a single hospital, which may restrict the generalizability of our findings. Additionally, we only considered PSS as a mediating variable; other factors may also influence the effect of depression on QOL in this patient group; for instances, patients’ psychological resilience, coping styles, rate of disease progression, and family financial status may have implications for depression and QOL, and the absence of these variables from this study may contribute to limitations in understanding the relationship between depression and QOL. Simultaneously, the unequal distribution of sociodemographic characteristics (e.g., significant gender imbalance) limited our ability to examine in depth the moderating effects of gender or other demographic factors (e.g., age, education) on the relationship between depression, PSS, and QOL. Furthermore, patients’ psychological processes may vary over time, which may potentially compromise the stability of study findings. For instance, patients may exhibit high levels of depression at the onset of chemotherapy, primarily due to anxieties concerning their illness and the treatment itself. However, as treatment advances and their understanding of the disease increases, these levels of depression may gradually subside. Nonetheless, cross-sectional study designs are inherently incapable of capturing such dynamic fluctuations. Therefore, to address the aforementioned limitations, future research could employ a multicenter, large - sample approach. This would involve collaborating with multiple hospitals to recruit a more diverse and representative cohort of patients undergoing chemotherapy for lung cancer. And the study design should incorporate additional variables, such as psychological resilience, coping strategies, disease progression rate, and family economic status. At the same time, targeted subgroup analyses (e.g., stratified by demographic variables such as gender, age, socioeconomic status, etc.) could be considered to more fully explore the mechanisms by which depression affects QOL; Lastly, a longitudinal study design should be utilized to track the dynamic changes in patients’ psychological processes over time, thereby providing a deeper understanding of the interplay between depression, PSS, and QOL.

## Data Availability

The raw data supporting the conclusions of this article will be made available by the authors, without undue reservation.
