# Supplementary material for: The impact of depression on the quality of life of lung cancer patients undergoing chemotherapy: mediating effects of perceived social support
Source: Front Psychiatry. 2025 Mar 21;16:1526217. doi: 10.3389/fpsyt.2025.1526217 (PMC11968671; doi:10.3389/fpsyt.2025.1526217)
Supplement: Supplementary file 2 [file Table2.docx]

This is a paper version of the scale used in the article " The impact of depression on the quality of life of lung cancer patients undergoing chemotherapy: mediating effects of perceived social support". We used this questionnaire in our survey of lung cancer patients undergoing chemotherapy.

**Quality of Life Scale and Related Factors Questionnaire for Lung Cancer Chemotherapy Patients**

Hello, thank you for taking time out of your busy schedule to fill out this questionnaire. We are researchers from Deyang People's Hospital, this is a questionnaire related to the quality of life of lung cancer chemotherapy patients, this survey is conducted in an anonymous way, the information you answer will be highly confidential and will not be disclosed to others, thank you for your cooperation and support!

**I. General information questionnaire**

**Guidance: The following questions are designed to investigate general information about you, please tick the appropriate box after each sub-question.**

1. Your gender ：□male □famale

2. Your age year

3. Number of children:

□none □one □two □3 and above

4. Marital status:

□married □singleton

5. Educational level:

□Primary and below □middle school □College and above

6. Religious beliefs

□yes □no

7. Occupational situation

□Work (including self-employment, freelancing, etc.) □retirement □unemployed

8. Monthly per capita household income :

□＜3000yuan □3000-4999 yuan □5000-9999 yuan □≥10000 yuan

9. current address

□municipalities □townships □villagers

10. Clinical Staging of Cancer

□Ⅰ □Ⅱ □Ⅲ □Ⅳ

11. Whether there is a combination of other diseases:

□yes □no

II. Chinese version of the Lung Cancer Therapeutic Function Rating Scale

**Guideline: Below is a survey of some questions related to your disease, please select the answer that matches your situation and tick the appropriate box after each sub-question.**

| I. Physiological status (PWB) | | | | | | |
| --- | --- | --- | --- | --- | --- | --- |
| Title number |  | Not at all | A little bit | A bit | Quite a bit | Very much |
| 1 | I lack energy. |  |  |  |  |  |
| 2 | I have vomiting. |  |  |  |  |  |
| 3 | I can't handle the daily life of a family. |  |  |  |  |  |
| 4 | I have pain. |  |  |  |  |  |
| 5 | I was plagued by toxic reactions from the treatment |  |  |  |  |  |
| 6 | Usually I'm weak. |  |  |  |  |  |
| 7 | I had to stay in bed. |  |  |  |  |  |
| II. Social/Family Well-Being (SWB) | | | | | | |
| Title number |  | Not at all | A little bit | A bit | Quite a bit | Very much |
| 1 | I got the closeness of a friend. |  |  |  |  |  |
| 2 | I get moral support from my family. |  |  |  |  |  |
| 3 | I got the support of my friends. |  |  |  |  |  |
| 4 | My family accepts my illness. |  |  |  |  |  |
| 5 | Satisfaction with communication between me and my family about my condition |  |  |  |  |  |
| 6 | I feel close to my partner (or the person I consider most important) |  |  |  |  |  |
| 7 | I'm happy with my sex life. |  |  |  |  |  |
| III. Emotional well-being (EWB) | | | | | | |
| Title number |  | Not at all | A little bit | A bit | Quite a bit | Very much |
| 1 | I'm sad. |  |  |  |  |  |
| 2 | I'm proud that I'm facing the disease. |  |  |  |  |  |
| 3 | Disappointed in my battle with the disease |  |  |  |  |  |
| 4 | I feel nervous. |  |  |  |  |  |
| 5 | I'm afraid of death. |  |  |  |  |  |
| 6 | I'm afraid I'm getting worse. |  |  |  |  |  |
| IV. Functioning well-being (FWB) | | | | | | |
| Title number |  | Not at all | A little bit | A bit | Quite a bit | Very much |
| 1 | I can work (including work done at home) |  |  |  |  |  |
| 2 | I work a lot. |  |  |  |  |  |
| 3 | I'm enjoying life at the moment. |  |  |  |  |  |
| 4 | I can accept my illness. |  |  |  |  |  |
| 5 | I sleep well. |  |  |  |  |  |
| 6 | I often do leisure activities and have fun with them |  |  |  |  |  |
| 7 | I am satisfied with my current quality of life |  |  |  |  |  |
| V. Lung Cancer Subscale (LCS) | | | | | | |
| Title number |  | Not at all | A little bit | A bit | Quite a bit | Very much |
| 1 | I feel short of breath. |  |  |  |  |  |
| 2 | I'm losing weight. |  |  |  |  |  |
| 3 | I'm thinking clearly. |  |  |  |  |  |
| 4 | I have a cough. |  |  |  |  |  |
| 5 | I suffer from hair loss. |  |  |  |  |  |
| 6 | I have a good appetite. |  |  |  |  |  |
| 7 | I feel tightness in my chest. |  |  |  |  |  |
| 8 | I'm breathing well. |  |  |  |  |  |
| 9 | I regret having smoked (non-smokers need not answer) |  |  |  |  |  |

**Ⅲ.Depression Self-Rating Scale**

**Guideline: The following is a survey of questions related to your psychological condition. Please select the answers that match your situation and tick the appropriate box after each sub-question.**

| Title number |  | Never or occasionally | Occasionally | Regular | Always |
| --- | --- | --- | --- | --- | --- |
| 1 | I feel emotionally frustrated and depressed. |  |  |  |  |
| 2 | I feel in the best mood in the morning. |  |  |  |  |
| 3 | I'm going to cry or want to cry. |  |  |  |  |
| 4 | I don't sleep well at night. |  |  |  |  |
| 5 | I eat as much as usual. |  |  |  |  |
| 6 | I have normal sexual function. |  |  |  |  |
| 7 | I feel weight loss. |  |  |  |  |
| 8 | I struggle with constipation. |  |  |  |  |
| 9 | My heart is beating faster than usual. |  |  |  |  |
| 10 | I feel tired for no reason. |  |  |  |  |
| 11 | My mind is clear as usual. |  |  |  |  |
| 12 | I don't find it difficult to do things as I usually do. |  |  |  |  |
| 13 | I'm having a hard time sitting down and staying calm. |  |  |  |  |
| 14 | I feel hopeful about the future. |  |  |  |  |
| 15 | I'm more easily irritated than usual. |  |  |  |  |
| 16 | I find it easy to decide what to do. |  |  |  |  |
| 17 | I feel like a useful and indispensable person. |  |  |  |  |
| 18 | I've had an interesting life. |  |  |  |  |
| 19 | Assuming that if I die, someone else will have a better life. |  |  |  |  |
| 20 | I still like my usual favorites. |  |  |  |  |

**Ⅳ、Perceived social support scale**

**Guideline: The following is a survey of questions related to your social support. Please choose the answers that match your situation and tick the appropriate box after each sub-question.**

| Title number |  | Super disagree | Strongly disagree | Slightly disagree | Neutral | Slightly agree | Strongly agree | Super agree |
| --- | --- | --- | --- | --- | --- | --- | --- | --- |
| 1 | There are people (leaders, relatives, coworkers) who are there for me when I have problems |  |  |  |  |  |  |  |
| 2 | I was able to share joys and sorrows with some people (leaders, relatives, coworkers) |  |  |  |  |  |  |  |
| 3 | My family has been able to help me in concrete ways. |  |  |  |  |  |  |  |
| 4 | I am able to get emotional help and support from my family when needed |  |  |  |  |  |  |  |
| 5 | Some people (leaders, relatives, coworkers) are a true source of comfort when I am in trouble |  |  |  |  |  |  |  |
| 6 | My friends can really help me. |  |  |  |  |  |  |  |
| 7 | I can count on my friends in times of trouble. |  |  |  |  |  |  |  |
| 8 | I can talk to my own family about my problems. |  |  |  |  |  |  |  |
| 9 | My friends can share my joys and sorrows. |  |  |  |  |  |  |  |
| 10 | There are certain people in my life (leaders, relatives, coworkers) who care about my feelings |  |  |  |  |  |  |  |
| 11 | My family willingly assists me in making decisions. |  |  |  |  |  |  |  |
| 12 | I can discuss my problems with my friends. |  |  |  |  |  |  |  |
